# Editorial

**Published:** 1861-07

**Authors:** 


### Editorial.

### DENTISTS IN THE ARMY.

ABOUT two years ago, there was a movement in some of the dental meetings of our country in reference to the appointment of dentists in the regular army. A committee was appointed to memorialize Congress upon the subject, and take such other steps as might seem practicable. Little or no effort, however, was made by that committee ; why, we do not know ; perhaps, upon a survey of the matter, the committee found the difficulties to be so great, as to render any effort in that direction impracticable. This movement was based upon the idea, that the welfare of the soldiers and all in army service required the services of the dentist. We believe this is a work which should have been prosecuted ; competent dentists should be attached to every army.

Dentistry is a subject to which physicians and surgeons do not give very close attention ; they too frequently overlook or disregard the diseases to which the teeth are subject, and their treatment ; and this when attempted, is usually confined to the extraction of the supposed offensive organ. More than this could not well be expected of them. Dentistry is one of the specialties of medical practice, that demands for its faithful performance, the undivided attention and effort of one person.

The effort of our associations in this matter was predicated upon the belief that the teeth of those in our armies should receive more attention than heretofore ; this can hardly be controverted. The teeth of soldiers by neglect, very frequently become diseased, and that at an early period of camp life. We are fully convinced that the soldiers of the regular army should have the services of good, efficient dentists, especially so far as hygienic, prophylactic, and remedial treatment is concerned. This, perhaps, should not include the expensive operation of filling, and the insertion of artificial teeth, though we think it would be well for the appointment to contemplate these, except a fee from the patient, which should amply cover the cost of material, and perhaps a nominal fee beyond this.

If those who have been in the regular army should have the services of a dentist, the claims of those who have recently entered its service will, in some respects, possess a stronger one. A very large proportion of these persons are from positions in society where they have been accustomed to receive such attention as their well-being demanded, and not least among these is the service of the dentist ; such persons will feel severely the loss of such attention. We think attention should be given at once to this subject by those having power and authority, and we shall, so far as opportunity may afford, bring the matter to their attention.

It is possible that such an appointment would be self-sustaining, or principally so ; it would certainly accomplish more in this respect than the general surgeon.

Another point worthy of consideration is, that in times of emergency such persons would be of great value to the general surgeon.

We shall have more to say upon this subject in future. T.

### THE INFLUENCE OF CAMP LIFE UPON THE TEETH.

The present disturbed and distracted condition of our country has caused a great and sudden change in the manner of life with many thousands of our countrymen. This is true of all those who have gone from pleasant and comfortable homes into camp life.

The change thus incurred will, doubtless, make its mark, and this in various ways. Some circumstances will be physically advantageous, while others will be adverse. Perhaps in no particular will the latter be more manifested, than upon the teeth of the soldiery. To these, the attention necessary to keep them in good condition is not given ; they are, as a general thing, wholly overlooked in regard to cleanliness, and also to diseased conditions, until they have become intolerable by the pain which they occasion. This want of attention results from two or three considerations. The excitement and novelty of the new position is, perhaps, at first a very general cause of neglect ; but the want of facilities, such as proper direction, tooth-brushes, dentifrices, tooth-picks, etc., constitutes the great difficulty lying in the way. These things should all be placed within the reach of every soldier ; but strange as it may seem, we have not been able to find anything of the kind, in a single sutler's establishment, in a large encampment.

We are familiar with cases, in which persons, before entering camp, were scrupulously careful in regard to the teeth, giving all attention, that nothing injurious should be permitted to remain upon them, who, after entering camp, were so inattentive as to permit vitiated food and a thick gumy mucus, and sordes, to accumulate upon the teeth in large quantities, exhibiting a most disgusting appearance, and producing decay and disease of the teeth with the greatest rapidity. This occurring to a great extent, no doubt, from the causes we have mentioned ; in addition to these, such persons, no doubt, feel that they can not avail themselves of the services of a dentist.

A large proportion of those who enter the army sustain great injury to the teeth in a short time. We remember numbers who went to the Mexican war, with sound and beautiful teeth, and returned within a year or two with them almost destroyed, and in some cases entirely so, and almost all much diseased. With the proper care, there is no excuse for this state of the teeth, even in an army, for they have the time, and should have the facilities afforded, to keep them in the best possible condition. T.

### MICROSCOPE.

The microscope purchased by the Mississippi Valley Association is complete in all its arrangements, and in working order. The specimens of sections of the teeth, exhibiting the dental structures, ordered by the last meeting of the Mississippi Valley Association, are now complete and ready for examination. These specimens are very fine, having been prepared with great care, with reference to the most perfect exhibitions of the structures of the dental tissues. The members of the society can have access to the instrument for the examination and study of these specimens, by calling at our office, at any time it may suit their convenience. We have in course of preparation a series of abnormal and pathological specimens which will be highly interesting. We invite the attention of our brethren to these also, as they afford a good subject for study. T.

### CORRECTION.

We have received a communication from Dr. W. M. Rogers, of Shelbyville, Ky., in which he states that he is incorrectly reported in the discussions of the Kentucky State Dental Association, in regard to the subject of caries. We will give his own remarks in regard to the matter:

"Your reporter misunderstood my remarks in the Kentucky Dental Association. I hold no such opinion, as that there is such a thing in dental pathology as internal decay. I can readily understand how my remarks might be misapprehended; but as they would not, probably, be of any general interest, I will simply beg that you will do me the favor to state in the Register, that I disclaim the doctrines imputed to me by the report, and endorse the editorial remarks of T."

By this it will be seen that Dr. R. sets himself right upon the subject of internal decay; and right glad we are of it. We thought the Doctor far too well posted to entertain any such idea, and we felt, when we read that report, that we had been mistaken. T.

### CREDIT.

Perhaps the Southern Dental Examiner would have given the Dental Register credit for the lengthy extract from its pages of Dr. Talbert's paper, read before the Kentucky State Dental Association, if its Editors had not forgotten or overlooked the matter. The custom is to give the credit; perhaps a new mode is to be introduced. T.

### MICROSCOPIC.

We request of our professional brethren, that they would send us any teeth of peculiar structure, or those having any unusual peculiarity which would be unfolded or brought out more fully by the microscope, to send them to us, and we will have them nicely mounted, so as to exhibit most fully any peculiarity they may possess. We hope this will receive the attention of our brethren, and that they will take advantage of this opportunity to save any rare specimens that they may find. T.

### CLOSURE OF THE PAROTID DUCT.

About six years ago, Mr. J. M. had an upper molar tooth, the fractured edges of which produced considerable irritation of the mucous membrane of the cheek. In a short time, inflammation was established, and the cheek began to swell. The tooth was extracted, but the swelling continued. There was, at the end of a year, very great enlargement, producing a very serious deformity ; and the general impression was that there was malignant disease of the parotid gland. At this stage the case came under my observation ; and, on examination, I found that the orifice of the parotid duct was closed. I made an incision, and endeavored, with probes, to re-open the duct, but failed. An isolated lower molar was found sensitive, on percussion, and slightly loosened, and I thought proper to extract it. When the tooth was extracted, a jet of purulent matter was thrown out of the patient's mouth, striking the window, in front of the operating chair, above the level of the patient's head. The quantity of matter discharged was about eight ounces. It had nothing of the appearance of healthy pus. In general, it was thin, watery and dark-colored, with large quantities of irregular masses, in appearance and texture like coagulated albumen. There was but little discharge of blood. After the discharge the parotid gland could be distinctly felt, and was found to be somewhat enlarged and indurated. An ordinary silver probe could be passsd its entire length, through the socket, in almost any direction.

The socket was kept open and the cavity of the abscess was regularly washed out with tepid water, and the case progressed favorably for some ten days, when violent inflammation set in, accompanied with great constitutional disturbance. Both local and general treatment were resorted to, and, as the inflammation subsided, an abscess opened through the mucous membrane, at the angle of its reflection from the cheek to the gum, and the opening through the socket was allowed to close.

For three years the patient has not been troubled with inflammatory or other unpleasant symptoms ; and the saliva is discharged through the new opening referred to, which seems to answer all the purposes of an artificial duct.

We have not thought it necessary to give a detail of the treatment pursued in this case, as minuteness is often more tedious than profitable. W.

### THE TEETH OF ARMY RECRUITS.

In the Manual for the Recruiting Service, we find the following given as sufficient causes for the rejection of applicants, viz :

Loss of the whole or part of either jaw-bone ;

Deformities of either jaw bone, interfering with mastication, speech, or the tearing of the cartridge ;

Anchylosis of the jaws ;

Loss of the incisor and canine teeth of both jaws.

"To this catalogue we may add *caries* or bad condition of a great many or most of the teeth ; it is necessary that the soldier should be able to chew his biscuit ; if he has lost some of the molar teeth, the others should be sound, as well as the gums that support them. Otherwise the jaws are exposed to frequent irritations ; to swellings under the influence of the slightest causes. In the inspection of substitutes, we should be very severe upon this point."*

To pronounce absolutely upon the number or description of the teeth that may be lost without disqualifying the recruit, would be a very difficult matter. All authors speak of this kind of defect but always in rather indefinite terms. The British Regulations, mention "loss of many teeth, or teeth generally unsound ;" Sir George Ballingall speaks of "extensive deficiency, particularly of the front teeth ;" the French, as above quoted, enumerate the loss of the whole of the canine and incisor teeth of both jaws.

The soldier requires a sufficient number of teeth in good condition, to enable him to masticate his food properly. Hard bread, tough beef, and salt pork, require good molars for this purpose. The incisor and canine teeth are not adapted to this end, *i. e*, without the aid of some of the molars. The soldier must again have teeth of some description, strong enough to tear his cartridge. This

* Aide Memoire.

is usually done with the incisor and canine teeth. But if the bicuspid and two of the molars in both jaws upon the right side remain and are sound, we think this may be done as conveniently as with the incisor and canine. The instructions for tearing the cartridge in the infantry tactics, merely prescribe that it is to be put between the teeth, without specifying the particular teeth by which it is to be torn.

If, then, the front teeth have been lost by accident, as sometimes happens, we should not reject the man on that account, provided the double teeth, or a sufficient number of them, remain sound in both jaws, and upon the right side. But if the front teeth have been lost from caries, and the double teeth are unsound to any extent, the man should be rejected. If the front teeth remain and the molars are gone, we think rejection is again demanded, because the man is evidently incapable of properly masticating the food he must subsist upon in the field.

"*Fetid breath*" is sometimes a reason for rejection. If merely a sign of temporary derangement of the digestive organs or the like, it is of no consequence. But if it depend upon extensive caries of the teeth, chronic ozœna, scorbutic, syphilitic, scrofulous, or mercurial cachexia, it demands rejection as well from its own offensiveness as from its being one of the indications of grave disqualifying disease.

If good teeth are so important for the soldier, would it not be well to adopt some means by which they can receive the attention necessary for their preservation? T.

### TEXTURE OF DENTINE.

The enamel of the teeth, as all know, is usually the hardest substance in the human frame. Lately, however, we met with a case which emphatically contradicted the rule. A lady of eighteen had lost the upper incisors by early decay. The bicuspids and molars were defective, but were filled. The enamel was defective on one of the cuspids. At one point the depression was so great as to afford a lodgment for foreign substances, and we concluded to form a cavity and fill. The border of enamel was easily dressed off, but our instruments refused to take hold of the dentine. The best drills —those that never refused to cut enamel—glided over it as if composed of soft iron. We surrendered, dressed off the border of enamel, to give a more uniform surface, and told the patient that, on further examination, it appeared that the tooth did not need filling.

